# Prediabetes Knowledge, Attitudes, Practices, and Risk Levels in the United Arab Emirates: A Cross-Sectional Study

**DOI:** 10.7759/cureus.82099

**Published:** 2025-04-11

**Authors:** Obada Al-Wawi, Hadeel Alameleh, Mazen Alhaj Ahmad, Fatima Al Shamsi, Laith Suwan, Jamila Bin Kowayer, Fadi Almaqableh, Amal Hussein, Nabil Sulaiman

**Affiliations:** 1 College of Medicine, University of Sharjah, Sharjah, ARE; 2 Family and Community Medicine, University of Sharjah, Sharjah, ARE; 3 Department of Medicine, Baker/IDI Heart and Diabetes Institute, Melbourne, AUS

**Keywords:** cross-sectional studies, diabetes, diabetes screening, gulf, health knowledge attitudes practices, middle east, prediabetes, prediabetes awareness, public health, risk factors

## Abstract

Background and aims

Prediabetes is a reversible state of mild hyperglycemia that significantly increases the risk of developing type 2 diabetes mellitus (T2DM). This study aimed to assess knowledge, attitudes, and practices (KAP), risk levels, and associated factors related to prediabetes among adults in the United Arab Emirates (UAE).

Methods

This cross-sectional study was conducted between February and March 2022 among adults (≥18 years) residing in the UAE, using non-probability convenience sampling. Data were collected through a self-administered online questionnaire using the Knowledge Attitude Practice - Prediabetes Assessment Questionnaire (KAP-PAQ) and the Saudi Diabetes Risk Score (SADRISC). Data analysis was performed using SPSS (IBM SPSS Statistics for Windows, IBM Corp., Version 28, Armonk, NY). Mann-Whitney U and Kruskal-Wallis tests were used for group comparisons, and Spearman correlation assessed associations. Participants with diabetes were excluded from the SADRISC-based risk analysis.

Results

A total of 414 participants completed the survey, 278 (67.1%) of whom were non-Emirati Arabs. Poor knowledge of prediabetes was observed in 269 (65%) of participants, and 348 (84.1%) reported poor to very poor practices. In contrast, 238 (57.5%) expressed positive to strongly positive attitudes. The SADRISC tool classified 105 (27.8%) non-diabetic participants as having a high risk for prediabetes or undiagnosed T2DM. Nearly half (203, 49.0%) were unaware that prediabetes progression to T2DM is preventable with intervention, and 244 (58.9%) had never checked their blood sugar levels. Higher knowledge levels were associated with better attitudes and practices.

Conclusion

This study reveals insufficient knowledge and poor health practices regarding prediabetes among UAE adults despite generally positive attitudes. A considerable proportion of participants were at high risk for prediabetes or undiagnosed T2DM. Nearly half were unaware that prediabetes progression is preventable. Despite national efforts, most had never checked their blood glucose levels. Targeted interventions, including more extensive awareness campaigns and screening programs, are crucial to improving knowledge, encouraging preventive behaviors, and enhancing early detection of prediabetes.

## Introduction

Prediabetes is a reversible condition characterized by blood glucose levels that are higher than normal but not high enough to be classified as diabetes. It is a high-risk state for developing type 2 diabetes mellitus (T2DM), a chronic disease that affected more than 537 million individuals worldwide in 2021 [1,2]. Without intervention, up to 70% of individuals with prediabetes may progress to T2DM, increasing their risk for cardiovascular disease, nephropathy, and neuropathy [1]. However, evidence shows that lifestyle changes and pharmacological interventions can reduce diabetes risk and even reverse prediabetes back to normoglycemia [3].

The burden of diabetes is particularly concerning in the United Arab Emirates (UAE), where 16.1% of adults aged 20 to 79 had diabetes in 2021 [2]. Additionally, a national health survey conducted in 2019 revealed that 16.2% of adults were prediabetic, highlighting the substantial at-risk population [4].

The Diabetes Prevention Program (DPP) trial, a landmark study, demonstrated that lifestyle modifications, such as increased physical activity, a healthy diet, and weight loss, reduced the risk of developing T2DM by 58%, whereas metformin use resulted in a 31% risk reduction. These findings emphasize the effectiveness of simple, low-cost interventions in delaying or preventing diabetes progression, underscoring the importance of early risk identification [3].

Public awareness plays a key role in diabetes prevention [5]. Studies from South India, China, and Japan have revealed poor awareness of prediabetes, with up to 93.7% of individuals unfamiliar with terms such as “prediabetes,” “impaired glucose tolerance,” “impaired fasting glucose,” or “borderline diabetes” [6]. While no similar studies have been conducted on the UAE’s general population, cultural and lifestyle differences may lead to unique knowledge, attitudes, and practices (KAP) patterns, highlighting the need for local data to guide national prevention strategies. Understanding these patterns is essential for preventing the progression from prediabetes to T2DM, as attitudes toward the condition are key in motivating individuals to adopt healthier lifestyle choices [7,8].

Risk assessment tools are crucial for identifying individuals at high risk of developing diabetes, particularly in high-prevalence regions such as the UAE. These tools help identify those who may benefit from early interventions, which have been shown to significantly reduce the incidence of T2DM [9]. While the Finnish Diabetes Risk Score (FINDRISC) is widely used in international studies, its applicability to Middle Eastern populations is limited. This study instead utilizes the Saudi Diabetes Risk Score (SADRISC), a Saudi-developed alternative tailored to the region’s genetic, dietary, and lifestyle factors, making it more suitable for diabetes risk assessment in the UAE [10,11].

Despite the high burden of diabetes and prediabetes in the UAE, no studies have specifically examined prediabetes-related KAP within the general population. This study aims to evaluate the KAP and risk levels of prediabetes among UAE adults and to identify factors associated with these domains.

Findings from this study can inform the development of targeted awareness campaigns and preventive strategies at the national level. By addressing specific knowledge gaps and evaluating public attitudes and practices, the study provides valuable insights that can help policymakers and healthcare providers design more effective screening and educational interventions. These findings may support the creation of culturally relevant, accessible initiatives to improve early detection and prevention of prediabetes and type 2 diabetes in the UAE.

## Materials and methods

Study design and setting

This cross-sectional study was conducted in the UAE between February and March of 2022. An online questionnaire (Appendices) was used and distributed through social media platforms such as WhatsApp, Facebook, and Instagram. The study was conducted in accordance with ethical guidelines and was approved by the Ethics and Research Committee of the University of Sharjah (REC-22-02-13-03-S). The Strengthening of the Reporting of Observational Studies in Epidemiology (STROBE) checklist was used in reporting this study [12].

Participants and data collection

The study included adults aged 18 years or older residing in the UAE who were either Arabic or English speakers with access to social media platforms. Participants who did not meet the inclusion criteria were excluded from the analysis. The survey was distributed via convenience sampling on social media platforms, and participants were encouraged to share it with others, initiating a snowball sampling process. The participants were provided with electronic informed consent before proceeding. The response rate was not calculated because the number of people to whom the questionnaire was sent was not tracked.

Sample size determination

The sample size for this study was calculated using Cochran's formula, which is based on the anticipated prevalence of the condition under investigation [13]. Given the lack of prior studies on prediabetes awareness among the general population in the UAE, a prevalence rate of 50% was assumed. A 95% confidence interval and a 5% margin of error were applied, resulting in a calculated minimum sample size of 385 adults. To account for potential non-responses or other errors, we increased the sample size target by 10%, increasing the final required sample size to 424 participants. 

Data sources/measurements

The questionnaire is composed of two distinct tools for data collection: the Knowledge Attitude Practice - Prediabetes Assessment Questionnaire (KAP-PAQ) and the Saudi Arabian Diabetes Risk Score (SADRISC) [11,14]. The KAP-PAQ consists of three components: knowledge, attitudes, and practices. The questionnaire was translated into Arabic and proofread by a specialist to ensure linguistic accuracy and cultural appropriateness. The questionnaire was piloted and adjusted accordingly.

The knowledge section covered topics such as prediabetes progression to diabetes, inheritance from T2DM, identification, diagnosis, management, and prognosis. This section consisted of 10 multiple-choice questions, each with four answer options with only one correct answer. Correct answers were awarded points, whereas incorrect answers received zero points. The scoring was structured as follows: questions 1, 2, 7, 8, 9, and 10 were worth one point each; question 3 was worth three points; questions 4 and 5 were worth two points each; and question 6 was worth four points. The total possible score was 17 points.

The attitudes section aimed to evaluate participants' feelings and beliefs about prediabetes and their attitudes toward lifestyle modifications. This section included 10 questions rated on a three-point Likert scale (strongly disagree, neither agree nor disagree, strongly agree). Positive responses were awarded one point, negative responses -1 point, and neutral responses zero points. The total score ranged from -10 to 10.

The practices section included 10 multiple-choice questions related to daily behaviors such as dietary intake, physical activity, sleep patterns, and meal frequency, in addition to health checkup frequency. Points were awarded on the basis of the frequency of practices, with zero points for the least favorable frequency, one point for acceptable practices, and two to four points for the most favorable frequencies. For questions 1, 2, and 8, four points were awarded for the most favorable frequency. The total possible score in this section was 26 points.

The SADRISC tool, a separate risk assessment instrument, is a non-invasive tool that is best suited to identify individuals with a high risk of prediabetes or undiagnosed T2DM. It consists of five variables: sex, age, waist circumference, history of hyperglycemia, and family history of diabetes. The SADRISC tool had a sensitivity of 69% and a specificity of 69% using a cut point of >6. To estimate participants' waist circumference, we employed pant size as a proxy measure due to the constraints imposed by the COVID-19 pandemic restrictions during the time of conducting the study.

Variables

The study analyzed several demographic and primary variables collected from participants. Continuous variables such as body mass index (BMI) were categorized as normal (<25 kg/m²), overweight (25-30 kg/m²), or obese (>30 kg/m²). Chronic illnesses were grouped into three categories: none, diabetes or hypertension, and other conditions. Other variables were also grouped during analysis as needed.

The knowledge scores were categorized as poor (<10), average (10-13), or good (14-17). The attitude scores were categorized as strongly negative (<0), negative (0-2), neutral (3-6), positive (7-8), or strongly positive (9-10). Practice scores were categorized as very poor (<6), poor (7-13), good (14-20), or very good (>20). SADRISC scores were categorized as low risk (0-6) or high risk (>6) of having prediabetes or undiagnosed T2DM.

Data analysis

The data were cleaned and analyzed using SPSS (IBM SPSS Statistics for Windows, IBM Corp., Version 28, Armonk, NY). During data cleaning, duplicate responses, implausible outliers, and data inconsistencies were removed to ensure the accuracy and integrity of the dataset. Descriptive statistics were used to summarize the demographic data and scores from the KAP-PAQ and SADRISC tools. Percentages and frequencies are reported for categorical variables. The normality of continuous variables was assessed using the Kolmogorov-Smirnov test and QQ plots. As the data were non-normally distributed, non-parametric tests (Mann-Whitney U and Kruskal-Wallis) were used for group comparisons. Spearman's rank correlation coefficient was used to examine associations between continuous KAP scores and the continuous SADRISC risk score, as well as correlations among the continuous KAP scores, with statistical significance set at p < 0.05.

To ensure the accuracy of the risk level correlations, participants who reported having diabetes were excluded from the analysis of SADRISC risk scores, so risk levels were only assessed for non-diabetic individuals. Confounders, such as demographic and lifestyle factors, were collected through the questionnaire; however, they were not specifically addressed in the analysis.

Bias

This study employed convenience and snowball sampling methods, which may have led to selection bias due to the overrepresentation of certain demographic groups active on social media. To mitigate this, recruitment was conducted across diverse social media platforms to capture a more representative sample of the UAE population.

Self-reporting bias was addressed through the use of the validated KAP-PAQ tool and the regionally developed SADRISC. Despite its limitations, SADRISC was selected for its practicality in an online setting, as it does not require laboratory measurements, making it feasible for a large-scale, remote study. The KAP-PAQ tool, which was originally validated for use in prediabetic individuals, was applied to the general population in this study. To address potential discrepancies, the questionnaire was piloted and adjusted based on participant feedback, although the performance of the KAP-PAQ in a broader population may differ from its intended use.

Additionally, the use of pant size as a proxy for waist circumference, due to the COVID-19 pandemic restrictions, may have introduced measurement bias. Conversion tables for US and EU pant sizes were provided to improve accuracy, but self-reported estimates are inherently less reliable than direct measurements. While efforts have been made to overcome these biases, the inherent limitations of the study design and tools should be considered when the findings are interpreted.

## Results

Participant characteristics

A total of 459 participants responded to the questionnaire, of whom 414 participants met the inclusion criteria and remained after data cleaning. A total of 278 (67.1%) were non-Emirati Arabs. Overall, 266 (64.3%) were female, and 161 (38.9%) were 18-24 years old. Currently or previously married participants made up 225 (54.3%) of the participants, and 223 (53.9%) had a diploma or bachelor's degree as their highest level of education. Most participants reported having no chronic illnesses (328, 79.2%). Additionally, 294 (71.0%) did not work or study in the medical field, and 226 (54.6%) reported not hearing or reading about prediabetes before. A total of 176 (42.5%) had a normal BMI (<25 kg/m²), and 178 (43.0%) reported having a first-degree relative with diabetes. A question about willingness to watch an educational video about prediabetes was included at the end of the questionnaire, and 296 (71.5%) were willing to watch it. Since diabetic participants were excluded from the risk level analysis, the characteristics of non-diabetic participants were reported separately. The full demographic details of all participants and non-diabetics are presented in Table 1.

**Table 1:** Participant characteristics (overall N = 414, non-diabetic n = 378) AED: United Arab Emirates Dirham

Participant characteristic	Overall N (%)	Non-diabetic n (%)
Sex		
Male	148 (35.7%)	132 (34.9%)
Female	266 (64.3%)	246 (65.1%)
Age		
18-24	161 (38.9%)	161 (42.6%)
25-34	73 (17.6%)	70 (18.5%)
35-44	79 (19.1%)	75 (19.8%)
45-54	74 (17.9%)	54 (14.3%)
>54	27 (6.5%)	18 (4.8%)
Nationality		
UAE national	96 (23.2%)	89 (23.5%)
Other Arab	278 (67.1%)	257 (68.0%)
Non-Arab	40 (9.7%)	32 (8.5%)
Marital status		
Single	189 (45.7%)	186 (49.2%)
Married (currently or previously)	225 (54.3%)	192 (50.8%)
Education		
High school or less	117 (28.2%)	112 (29.6%)
Diploma or bachelor’s degree	223 (53.9%)	202 (53.4%)
Postgraduate degree	74 (17.9%)	64 (16.9%)
Total house income per month		
Less than 5,000 AED	56 (13.5%)	53 (14.0%)
5,001 to 10,000 AED	94 (22.7%)	83 (22.0%)
10,001 to 15,000 AED	73 (17.6%)	66 (17.5%)
More than 15,000 AED	191 (46.1%)	176 (46.6%)
Smoking		
Never smoked	321 (77.5%)	292 (77.2%)
Current smoker	73 (17.6%)	67 (17.7%)
Ex-smoker	20 (4.8%)	19 (5.0%)
Chronic illnesses		
None	328 (79.2%)	328 (86.8%)
Diabetes and/or hypertension	63 (15.2%)	27 (7.1%)
Other	23 (5.6%)	23 (6.1%)
Medical Insurance		
No	98 (23.7%)	89 (23.5%)
Yes	316 (76.3%)	289 (76.5%)
Working/studying in the medical field		
No	294 (71.0%)	264 (69.8%)
Yes	120 (29.0%)	114 (30.2%)
Heard or read about prediabetes before		
No	226 (54.6%)	211 (55.8%)
Yes	188 (45.4%)	167 (44.2%)
Body mass index (BMI)		
Normal <25 kg/m^2^	176 (42.5%)	171 (45.2%)
Overweight 25-30 kg/m^2^	136 (32.9%)	122 (32.3%)
Obese >30 kg/m^2^	102 (24.6%)	85 (22.5%)
Waist circumference		
<94 cm (men)/<80 cm (women)	232 (56.0%)	220 (58.2%)
94-102 cm (men)/80-88 cm (women)	122 (29.5%)	107 (28.3%)
>102 cm (men)/>88 cm (women)	60 (14.5%)	51 (13.5%)
Past history of hyperglycemia (in a medical checkup, during an illness or pregnancy)		
No	343 (82.9%)	335 (88.6%)
Yes	71 (17.1%)	43 (11.4%)
Family history of diabetes		
No family history	104 (25.1%)	102 (27.0%)
Second-degree relative	132 (31.9%)	126 (33.3%)
First-degree relative	178 (43.0%)	150 (39.7%)
Willing to watch an educational video about prediabetes		
No	118 (28.5%)	112 (29.6%)
Yes	296 (71.5%)	266 (70.4%)

Risk levels

**Figure 1:**
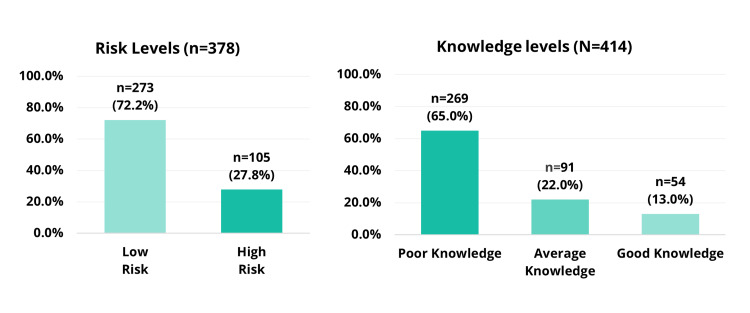
Risk levels of having prediabetes or type 2 diabetes mellitus in non-diabetic participants according to the SADRISC and knowledge levels among all participants SADRISC: Saudi Diabetes Risk Score

Risk levels were determined using the SADRISC tool, which identifies individuals at high risk for prediabetes or undiagnosed T2DM. Scores ranged from 0 to 13, with a median score of four (IQR 2-7). Based on their scores, 105 (27.8%) of non-diabetic participants were categorized as high-risk, whereas 273 (72.2%) were categorized as low-risk. The distribution of risk levels is illustrated in Figure 1.

Knowledge 

The median knowledge score was seven (IQR 3-11). A considerable proportion of participants demonstrated poor knowledge of prediabetes (269, 65.0%), whereas 91 (22.0%) had average knowledge, and only 54 (13.0%) exhibited good knowledge. These results indicate a general lack of awareness about prediabetes, even among individuals with higher levels of education. Notably, 44 (10.6%) participants scored 0 points on the knowledge assessment. Figure 1 presents the distribution of knowledge levels.

Most participants had limited knowledge regarding the identification and diagnosis of prediabetes. Correct responses were recorded for fasting blood glucose levels indicating prediabetes (123, 29.7%), prediabetes progression to T2DM (128, 30.9%), the likelihood of developing prediabetes with a family history of T2DM (138, 33.3%), the importance of insulin level testing (123, 29.7%), and blood tests being the best method for diagnosing prediabetes (158, 38.2%). Additionally, only 184 (44.4%) correctly identified HbA1c as the preferred test for measuring mean blood glucose levels.

While knowledge of prediabetes identification and diagnosis was generally low, participants demonstrated better awareness of its management. The majority were aware of the benefits of weight loss (306, 73.9%), diet (261, 63.0%), and exercise (297, 71.7%) in managing prediabetes. Finally, 326 (78.7%) correctly identified both diet control and exercise as the optimal strategies for managing the condition.

Attitudes

**Figure 2:**
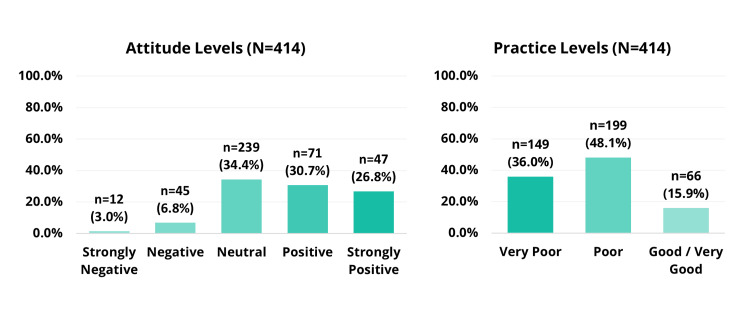
Attitudes and practice levels toward prediabetes

The attitudes toward prediabetes were generally positive, with a median score of 7 (IQR 5-9). The majority of participants (238, 57.5%) expressed positive to strongly positive attitudes, whereas 142 (34.3%) reported neutral attitudes, and only 34 (8.2%) held negative to strongly negative attitudes. Figure 2 illustrates the distribution of participants' attitudes toward prediabetes and its management.

A key finding was that 203 (49.0%) of participants were unaware that prediabetes progression to T2DM can be prevented if blood glucose levels are controlled. This misconception persisted despite widespread recognition of the importance of blood glucose control and lifestyle modifications in managing prediabetes.

Notably, 341 (82.4%) of participants acknowledged that actions could be taken to manage prediabetes. Additionally, 368 (88.9%) recognized the importance of maintaining blood glucose levels near normal in prediabetic individuals. However, 156 (37.7%) believed that controlling blood glucose is difficult for prediabetic individuals.

The participants overwhelmingly recognized the importance of lifestyle education, with 379 (91.5%) agreeing that individuals with prediabetes should be taught about lifestyle modifications. Importantly, 330 (79.7%) agreed that prediabetes is often ignored by society, and 353 (85.3%) recognized the importance of public education in prediabetes awareness. Additionally, 368 (88.9%) participants valued family support in managing prediabetes. Furthermore, 315 (76.1%) believed that individuals with prediabetes could still lead a normal life. Encouragingly, 254 (61.4%) disagreed with the misconception that prediabetes occurs only in "unlucky" individuals.

Practices 

The participants reported various health-related practices, including dietary habits, physical activity, sleep patterns, meal frequency, and health monitoring behaviors. The total practice score was 26, with a median score of eight (IQR 5-11), indicating overall poor practices. According to the practice score categories, 149 (36.0%) of participants demonstrated very poor practices, 199 (48.1%) had poor practices, and only 66 (15.9%) exhibited good to very good practices. Figure 2 illustrates the distribution of participants' practice levels.

With respect to dietary habits, 130 (31.4%) of the respondents reported "almost never" consuming sweetened beverages, whereas 146 (35.3%) consumed fiber-rich foods three to four times per week. In contrast, high-fat food consumption was more common, with 161 (38.9%) consuming such foods one to two times per week. The meal frequency patterns revealed that 143 (34.5%) participants skipped meals one to two times per week. Additionally, 147 (35.5%) reported engaging in distracted eating - defined as eating while watching TV, using a mobile phone, or reading - during every meal, and 173 (41.8%) spent more than six hours per day on screens.

In terms of physical activity, only 120 (29.0%) participants engaged in exercise for three to six hours per week. The sleep patterns revealed that 131 (31.6%) slept less than six hours per night, one to two times per week.

Health monitoring habits were suboptimal, with 244 (58.9%) of participants never checking their blood sugar levels, whereas 84 (20.3%) reported doing so once every six months or yearly. Similarly, 206 (49.8%) never checked their cholesterol levels, whereas 163 (39.4%) reported checking it once or more in two years.

Associations with risk levels

Several factors were significantly associated with risk levels, as determined by the Kruskal-Wallis and Mann-Whitney U tests (see Table 2). These included nationality, with non-Emirati Arabs having a higher mean rank (M = 197.37, p = 0.049), marital status, where those currently or previously married exhibited a higher mean rank (M = 241.93, p < 0.001), and education level, with postgraduate degree holders showing the highest mean rank (M = 251.93, p < 0.001). A total monthly household income of 5,001-10,000 AED (United Arab Emirates Dirham) was also associated with increased risk (M = 208.17, p = 0.026). Smoking status, including current or former smokers, was correlated with higher risk (M = 210.51, p = 0.041). Additionally, health conditions such as hypertension (M = 310.44, p < 0.001) and obesity (BMI >30 kg/m², M = 285.55, p < 0.001) were strongly associated with elevated risk levels. Notably, participants at higher risk were more likely to express willingness to watch an educational video about prediabetes (M = 205.04, p < 0.001).

**Table 2:** Comparisons of SADRISC mean scores and demographic characteristics (n = 378) *p < 0.05; ^a^Mann-Whitney U test; ^b^Kruskal-Wallis test AED: United Arab Emirates Dirham; SADRISC: Saudi Diabetes Risk Score

Demographic	n	Risk level mean rank	Test statistic	p-value
Emirate				
Sharjah	178	191.25	H = 3.164^b^	0.367
Dubai	86	179.62		
Abu Dhabi	55	209.74		
Other emirates	59	179.75		
Nationality				
UAE National	89	180.74	H = 6.050^b^	0.049*
Other Arab	257	197.37		
Non-Arab	32	150.70		
Marital status				
Single	186	135.38	Z = 9.566^a^	0.001*
Married (currently or previously)	192	241.93		
Education				
High school or less	112	150.47	H = 35.904^b^	0.001*
Diploma or bachelor’s degree	202	191.36		
Postgraduate degree	64	251.93		
Total house income per month				
Less than 5,000 AED	53	153.20	H = 9.301^b^	0.026*
5,001 to 10,000 AED	83	208.17		
10,001 to 15,000 AED	66	180.74		
>15,000 AED	176	194.91		
Smoking				
Never smoked	292	183.31	Z = 2.048^a^	0.041*
Current or ex-smoker	86	210.51		
Chronic illnesses				
Hypertensive	27	310.44	Z = 6.024^a^	0.001*
Non hypertensive	351	180.20		
Medical insurance				
No	89	189.25	Z = 0.025^a^	0.980
Yes	289	189.58		
Working/studying in the medical field				
No	264	192.61	Z = 0.851^a^	0.395
Yes	114	182.29		
Heard or read about prediabetes before				
No	211	180.29	Z = 1.860^a^	0.063
Yes	167	201.14		
Body mass index (BMI)				
Normal <25 kg/m^2^	171	121.73	H = 142.140^b^	0.001*
Overweight 25-30 kg/m^2^	122	217.57		
Obese >30 kg/m^2^	85	285.55		
Attitudes toward prediabetes reversibility with intervention				
Unaware	187	188.08	Z = 0.158^a^	0.801
Aware	191	190.89		
Willingness to watch an educational video				
No	112	152.60	Z = 4.300^a^	0.001*
Yes	266	205.04		

On the other hand, risk levels were not significantly associated with the emirate of residence, having medical insurance, having heard or read about prediabetes before, or being aware of prediabetes reversibility. Additionally, individuals working in the medical field and those outside it had comparable risk levels.

Associations with knowledge levels

Table 3 presents the full details of the associations between KAP toward prediabetes and various demographic factors. The mean knowledge scores for prediabetes across demographic characteristics were compared using the Mann-Whitney U test for two independent groups and the Kruskal-Wallis test for more than two groups.

**Table 3:** Comparisons of KAP mean scores and demographic characteristics (N = 414) *p < 0.05; ^a^Mann-Whitney U test; ^b^Kruskal-Wallis test AED: United Arab Emirates Dirham; KAP: knowledge, attitudes, and practices; NA: not applicable. Correlation was not computed because this variable is a constituent of the attitudes score, making the correlation redundant.

Demographic	n	Knowledge			Attitudes			Practices		
		Mean rank	Test statistic	p-value	Mean rank	Test statistic	p-value	Mean rank	Test statistic	p-value
Sex										
Male	148	192.03	Z = 1.967^a^	0.049*	199.75	Z = 0.992^a^	0.321	195.13	Z = 1.573^a^	0.116
Female	266	216.11			211.81			214.39		
Age										
18-24	161	176.74	H = 22.274^b^	0.001*	191.21	H = 11.180^b^	0.025*	158.90	H = 58.890^b^	0.001*
25-34	73	202.74			221.66			196.32		
35-44	79	233.72			209.23			242.74		
45-54	74	244.1			238.78			272.10		
>55	27	226.72			175.59			247.35		
Nationality										
UAE National	96	189.74	H = 5.504^b^	0.064	189.86	H = 3.179^b^	0.204	192.72	H = 2.852^b^	0.240
Other Arab	278	208.66			214.46			209.51		
Non-Arab	40	242.08			201.46			229.04		
Marital status										
Single	189	187.30	Z = 3.155^a^	0.002*	198.62	Z = 1.395^a^	0.163	164.35	Z = 6.740^a^	0.001*
Married (currently or previously)	225	224.47			214.96			243.74		
Education										
High school or less	117	189.04	H = 9.807^b^	0.007*	218.70	H = 3.799^b^	0.150	183.61	H = 7.943^b^	0.019*
Diploma or bachelor’s degree	223	205.07			197.01			212.19		
Postgraduate degree	74	244.01			221.41			231.19		
Total house income per month										
Less than 5,000 AED	56	154.30	H = 14.787^b^	0.002*	165.00	H = 9.278^b^	0.026*	169.29	H = 9.111^b^	0.028*
5,001 to 10,000 AED	94	213.13			205.38			230.11		
10,001 to 15,000 AED	73	200.54			211.04			208.29		
>15,000 AED	191	222.98			219.65			207.27		
Smoking										
Never smoked	321	211.72	Z = 1.337^a^	0.181	212.73	Z = 1.666^a^	0.096	218.68	Z = 3.540^a^	0.001*
Current or ex-smoker	93	192.92			189.44			168.91		
Chronic illnesses										
None	328	193.57	H = 25.293^b^	0.001*	204.97	H = 1.081^b^	0.583	199.84	H = 11.155^b^	0.004*
Diabetes and/or hypertension	63	275.79			221.81			253.49		
Other	23	219.13			204.41			190.74		
Medical insurance										
No	98	194.88	Z = 1.197^a^	0.231	206.63	Z = 0.083^a^	0.934	212.42	Z = 0.467^a^	0.641
Yes	316	211.41			207.77			205.97		
Working/studying in the medical field										
No	294	178.80	Z = 7.656^a^	0.001*	195.76	Z = 3.151^a^	0.002*	211.55	Z = 1.082^a^	0.279
Yes	120	227.81			236.27			197.57		
Heard or read about prediabetes before										
No	226	157.04	Z = 9.428^a^	0.001*	183.96	Z = 4.424^a^	0.001*	196.85	Z = 1.990^a^	0.047*
Yes	188	268.16			235.79			220.30		
Body mass index (BMI)										
Normal <25 kg/m^2^	176	194.63	H = 3.561^b^	0.169	205.53	H = 1.572^b^	0.456	185.31	H = 10.708^b^	0.005*
Overweight 25-30 kg/m^2^	136	216.71			217.08			226.29		
Obese >30 kg/m^2^	102	217.43			198.12			220.74		
Family history of diabetes										
No family history	104	182.14	H = 15.230^b^	0.001*	207.33	H = 0.087^b^	0.957	208.31	H = 0.055^b^	0.973
Second-degree relative	132	192.43			209.87			205.50		
First-degree relative	178	233.49			205.85			208.51		
Attitudes toward prediabetes reversibility with intervention										
Unaware	203	155.20	Z = 40.198^a^	0.001*	NA	NA	NA	197.71	Z = 1.636^a^	0.102
Aware	211	223.08			NA			216.92		
Willingness to watch an educational video										
No	296	191.89	Z = 1.680^a^	0.093	196.16	Z = 1.227^a^	0.220	178.73	Z = 3.096^a^	0.002*
Yes	118	213.72			212.02			218.97		

The results revealed significant differences in knowledge scores across age groups, with respondents aged 45-54 years having the highest mean rank (M = 244.1, p < 0.001) compared with other age groups. Marital status was also a significant factor, as those who were currently or previously married had a higher mean rank (M = 224.47, p = 0.002). Similarly, respondents with a postgraduate degree had the highest mean rank for prediabetes knowledge (M = 244.01, p = 0.007) compared with those with lower educational levels.

Income was another significant factor, with respondents earning less than 5,000 AED per month having a significantly lower mean rank (M = 154.30, p = 0.002). Those with chronic illnesses such as diabetes and/or hypertension had much higher knowledge scores (M = 275.79, p < 0.001). As expected, respondents working or studying in the medical field had significantly higher knowledge scores (M = 227.81, p < 0.001), as did those who had previously heard or read about prediabetes (M = 268.16, p < 0.001). A higher mean rank for knowledge was also observed among respondents with a family history of diabetes, specifically those with first-degree relatives affected (M = 233.49, p < 0.001). Additionally, those aware that prediabetes is reversible with intervention demonstrated significantly higher knowledge scores (M = 223.08, p < 0.001).

No significant differences in knowledge levels were found across nationalities, BMI categories, or individuals with or without medical insurance. Furthermore, participants' knowledge did not influence their willingness to watch an educational video about prediabetes.

Associations with attitude levels

The results revealed significant differences in attitudes toward prediabetes across various demographic characteristics. The respondents aged 45-54 years had a higher mean attitude score (M = 238.78, p = 0.025). Those with a lower total household income (<5,000 AED) exhibited more negative attitudes toward prediabetes (M = 165.00, p = 0.026). Respondents working or studying in the medical field displayed significantly more positive attitudes toward prediabetes (M = 236.27, p < 0.001), as did those who had previously heard or read about prediabetes (M = 235.79, p < 0.001). Attitude levels were not significantly influenced by sex, nationality, educational level, personal history of chronic disease, or family history of diabetes.

Associations with practice levels

The results revealed significant differences in prediabetes-related practices across several demographic characteristics. The respondents aged 45-54 years exhibited better practices (M = 272.10, p < 0.001), as did those who were currently or previously married (M = 243.74, p < 0.001). The respondents with a postgraduate degree demonstrated better practices (M = 231.19, p = 0.019), whereas those with a monthly household income of less than 5,000 AED had poorer practice scores (M = 169.29, p = 0.028). Current or ex-smokers had significantly poorer practices (M = 168.91, p < 0.001). The respondents with chronic illnesses, including diabetes and/or hypertension, exhibited better practices (M = 253.49, p = 0.004). Those who had previously heard or read about prediabetes demonstrated better practices (M = 220.30, p = 0.047). Additionally, respondents with a BMI in the overweight range (25-30 kg/m²) had higher practice scores (M = 226.29, p = 0.005). Those willing to watch an educational video on prediabetes also showed improved practices (M = 218.97, p = 0.002).

In contrast, no significant differences in practice levels were observed based on sex or nationality. Having medical insurance did not influence lifestyle behaviors or the frequency of health checkups. A family history of diabetes was not associated with better or worse practice levels. Additionally, awareness of the reversibility of prediabetes through intervention did not lead to improved practices.

Associations in the KAP domain

Spearman's rho test was performed to explore the relationships between KAP and risk levels (Table 4). The results revealed a significant positive correlation of medium effect size between knowledge and attitude (r_s_ = 0.397, p < 0.001), indicating that individuals with better knowledge tend to have more favorable attitudes. Additionally, knowledge showed a significant positive correlation with practice (r_s_ = 0.164, p < 0.001), although with a small effect size, suggesting a weaker association.

**Table 4:** Correlation between KAP and risk using Spearman's rho test (N = 414) *p < 0.05; ^a^small effect size; ^b^medium effect size; ^c^large effect size KAP: knowledge, attitudes, and practices

Variable	Spearman’s rho (r_s_)	p-value
Knowledge and attitudes	0.397^b^	<0.001*
Knowledge and practices	0.164^a^	<0.001*
Attitude and practices	0.117^a^	0.017*
Risk and knowledge	0.190^a^	<0.001*
Risk and practices	0.227^a^	<0.001*

Furthermore, a positive relationship of small effect size was found between attitudes and practices (r_s_ = 0.117, p = 0.017), suggesting that individuals with more positive attitudes toward prediabetes management are slightly more likely to engage in better health practices. Risk was positively correlated with knowledge (r_s_ = 0.190, p < 0.001) and practices (r_s_ = 0.227, p < 0.001), both with small effect sizes.

## Discussion

Our study, which employed a validated tool (KAP-PAQ) and a regionally developed risk prediction tool (SADRISC) via an online survey distributed on social media, provides valuable insights into prediabetes risk, KAP among UAE adults. Most participants were unaware of prediabetes, as they had never heard or read about it before. More than one-fourth of the participants were at elevated risk for prediabetes or T2DM. Despite this, the findings indicate a widespread lack of knowledge about prediabetes, even among individuals with higher levels of education. While attitudes ranged from neutral to positive, practices were found to be significantly lacking.

However, it is important to note that our cross-sectional design and non-random sampling strategy may have introduced selection bias. In addition, the reliance on self-reported data could have led to measurement inaccuracies. Despite these limitations, our culturally adapted questionnaire, piloted for clarity and reliability, along with adherence to ethical and STROBE guidelines, helped ensure internal consistency, although the generalizability of the findings is limited to a population active on social media.

The UAE population exhibits several well-established risk factors for diabetes and prediabetes, including obesity, a family history of diabetes, and dyslipidemia. A study by Hamoudi et al. identified these factors as key contributors to prediabetes risk among various ethnic groups in the UAE [15]. Obesity is particularly prevalent, with 35% of adults classified as obese and 32% as overweight. Coupled with sedentary behavior, these factors contribute to a high rate of metabolic syndrome, which affects approximately 37.4% of the population [16]. As a result, the likelihood of developing diabetes or prediabetes is significantly increased. Our findings are consistent with these statistics, as 27.8% of non-diabetic participants were classified as high-risk for prediabetes or undiagnosed T2DM. This high percentage may be explained by the fact that risk scores identify not only individuals who are likely to have prediabetes or T2DM but also those at high risk of developing these conditions in the future.

Surprisingly, greater risk was associated with better knowledge and practices. However, health practices are influenced by multiple factors. One possible explanation is that individuals who perceive themselves to be at greater risk for diabetes may be more motivated to adopt preventive measures, such as attempting weight loss. However, this perception does not necessarily correlate with meeting physical activity guidelines or achieving weight loss [17]. Additionally, risk perception can drive people to actively seek out health-related information, which may further contribute to their engagement in preventive behaviors [18].

Knowledge plays a crucial role in preventing prediabetes and diabetes, as it empowers individuals to make informed decisions about their health and manage risk factors effectively [19]. Studies have shown that patients with a better understanding of their medications and self-care practices tend to have better glycemic control [20]. However, our population showed a significant gap in this area, with two-thirds (65%) demonstrating poor knowledge about prediabetes. This finding is consistent with a study in South India, where 90% of prediabetic individuals had inadequate knowledge [5]. In contrast, a study from Saudi Arabia reported that 87.1% of participants were well-informed about prediabetes [19]. These regional differences may be attributed to variations in public health initiatives, awareness campaigns, or the accessibility of healthcare education.

Higher knowledge levels about prediabetes and diabetes are strongly correlated with better attitudes and practices, as shown in previous studies [21]. In Mohsina et al.’s study, an educational program led to significant improvements in knowledge, attitudes, and practice scores [5]. Consistent with these findings, our study uncovered a significant positive correlation between knowledge and attitudes (r_s_ = 0.397, p < 0.001) and between knowledge and practices (r_s_ = 0.164, p < 0.001). These results emphasize the vital role of awareness in promoting positive attitudes, encouraging healthier behaviors, and supporting diabetes prevention efforts.

Attitudes toward prediabetes vary across populations. For example, in South India, only 1.9% of newly diagnosed prediabetic individuals had strongly positive attitudes, whereas 54% exhibited neutral attitudes [5]. Similarly, in Iran, almost half of the participants held negative views toward the lifestyle modifications necessary for managing prediabetes [21]. In contrast, our study revealed that the population exhibited predominantly neutral to strongly positive attitudes toward prediabetes, highlighting potential receptiveness to awareness programs. Importantly, this openness presents a valuable opportunity to implement awareness initiatives, as the low prevalence of negative preconceived notions suggests that the population is likely to be receptive to educational efforts. Furthermore, promoting positive attitudes may be particularly beneficial, as our findings indicate a significant correlation between positive attitudes and better practices (r_s_ = 0.164, p < 0.001).

A crucial part of managing prediabetes is being aware of the effect of glycemic control on the progression to T2DM. Alarmingly, 49.0% of participants were unaware that prediabetes can be reversed with simple medications and lifestyle modifications and that the progression to diabetes can be prevented [3]. This number is even higher than in a study conducted in Saudi Arabia, where 26.1% were unaware of the reversibility of the condition, highlighting a wide gap in knowledge [22].

Another significant challenge is the belief that controlling blood sugar levels in prediabetes is difficult. More than a third of participants held this belief, which is consistent with findings from Saudi Arabia, where 43% of individuals shared similar attitudes [22]. This perception is not necessarily caused by a lack of knowledge, as many individuals with diabetes understand their condition and the importance of controlling blood sugar levels. However, they often do not prioritize diabetes management due to other life stressors and responsibilities [23]. Hence, further qualitative research is necessary to identify the perceived barriers to diabetes and prediabetes self-management, as well as aspects that could motivate individuals to be more proactive in managing their condition.

The UAE population exhibits a predominantly sedentary lifestyle with low levels of physical activity. Additionally, their diet is characterized by a high intake of carbohydrates, including refined grains and high-calorie snacks. There is also a high consumption of sweetened beverages, which contribute significantly to daily caloric intake, accounting for 8-14% of total calories [24]. All of these factors are reflected in our study, where more than 80% of participants demonstrated poor to very poor lifestyle practices. Notably, there were no significant differences in practice levels or frequency of health checkups between participants with medical insurance and those without.

Our participants demonstrated poor knowledge of prediabetes and T2DM definitions and diagnostics, which was reflected in their practices. Of our population, 58.9% reported never having checked their blood sugar levels. Similar patterns have been reported in Jordan and Saudi Arabia, where 52.8% and 66.5% of individuals, respectively, almost never checked their blood sugar [22,25]. Given that our population is at high risk, many are not undergoing the necessary tests to detect the condition while still asymptomatic, missing a crucial window for early intervention.

The UAE has implemented multiple national initiatives to screen for diabetes and its risk factors [16,26]. However, despite these efforts, our findings suggest that screening programs are underutilized. This highlights the need to expand outreach and accessibility, ensuring that routine screening becomes more widely adopted.

The study findings highlight a need for targeted public health interventions to enhance prediabetes awareness and prevention. With 79.7% of participants believing that prediabetes is ignored by society, there is a strong demand for educational campaigns focusing on lifestyle modifications and blood sugar control [5,27]. Overall, the neutral to positive attitudes observed among participants suggest that the population is open to such initiatives. This openness is further supported by the fact that 71.5% of participants expressed interest in learning more about prediabetes, as evidenced by their agreement to watch an educational video.

To address these gaps, tailored awareness campaigns should focus on educating at-risk populations about prediabetes risks, correcting common misconceptions, equipping individuals with practical prevention and management strategies, and educating them about the effectiveness and importance of these interventions. Given that all study participants were social media users, employing the use of multimedia and digital platforms can significantly enhance outreach and engagement [28]. Short, engaging videos and interactive content can effectively convey crucial health messages, ensuring accessibility and retention of information [29].

## Conclusions

In this study of adults residing in the UAE, over one-fourth of participants were found to be at risk of developing prediabetes or T2DM, yet nearly half were unaware that progression from prediabetes to diabetes could be prevented. Additionally, knowledge about prediabetes and healthy lifestyle practices were insufficient. While attitudes were mostly neutral to strongly positive, practices remained poor, with almost two-thirds of participants never having checked their blood sugar levels. Despite existing national screening efforts, participation remains low, highlighting the need to improve accessibility and outreach.

To address these gaps, awareness campaigns and screening programs should focus on improving KAP while increasing participation in preventive medicine. Given the high level of engagement with social media, digital health strategies, including short educational videos and interactive content, could enhance outreach and accessibility. Future research should focus on measuring the effect of educational campaigns on the level of KAP toward prediabetes in the UAE population.

## Appendices

**Table 5:** English version of the questionnaire ^*^correct answer; ^#^one point awarded for agree and strongly agree; ^$^one point awarded for disagree and strongly disagree; ^§^acceptable practices; ^†^most favorable practices AED: United Arab Emirates Dirham

Section name	Item number	Questions/instructions	Choices
Demographics	D1	Sex	Male
			Female
	D2	Age group	Below 18 years old
			18-24 years old
			25-34 years old
			35-44 years old
			45-54 years old
			55-64 years old
			65 years old or more
	D3	Nationality	UAE national
			Other Arab
			Non-Arab
	D4	Social status	Single
			Married
			Divorced
			Widowed
	D5	In which Emirate do you live?	Sharjah
			Dubai
			Abu Dhabi
			Fujairah
			Ajman
			Ras al-Khaimah
			Umm al-Quwain
			I don’t live in the UAE
	D6	Highest level of education acquired	Primary school
			Secondary school
			High school
			Diploma
			Bachelor’s degree
			Post-graduate degree
	D7	What is your total household income per month?	Less than 5,000 AED
			5,001 to 10,000 AED
			10,001 to 15,000 AED
			More than 15,000 AED
	D8	Do you smoke?	Never smoked
			Yes, current smoker
			Ex-smoker
	D9	Which of the following long-term medical conditions do you have? (select all that apply)	None
			Heart disease
			High blood pressure
			Diabetes
			Asthma
			Liver disease
			Cancer
			Immunodeficiency
			Long-term kidney disease
	D10	Do you study or work in the medical field?	Yes
			No
	D11	Do you have medical insurance?	Yes
			No
	D12	Have you ever heard or read about prediabetes before completing this questionnaire?	Yes
			No
Risk level of prediabetes	R1	(For males) What is your waist circumference? (Measured below the ribs, usually at the level of the navel) - choose "I don't know" if you don't know the answer - (for females) pick the last choice	Less than 94 cm
			94-102 cm
			More than 102 cm
			I don’t know
			(Select this if you’re a female to skip the question)
	R2	(For females) What is your waist circumference? (Measured below the ribs, usually at the level of the navel) - choose "I don't know" if you don't know the answer - (for males) pick the last choice	Less than 80 cm
			80-88 cm
			More than 88 cm
			I don’t know
			(select this if you’re a male to skip the question)
	R3	(For males) What is the pant size that you usually wear (US)? - (for females) pick the last choice	34 or less
			35 - 37
			38 or more
			(Select this if you’re a female to skip the question)
	R4	(For females) What is the pant size that you usually wear (EU)? - (for males) pick the last choice	43 or less
			44-47
			48 or above
			(Select this if you’re a male to skip the question)
	R5	Have you ever been found to have high blood glucose (e.g., in a health examination, during an illness, during pregnancy)?	Yes
			No
	R6	Have any of the members of your immediate family or other relatives been diagnosed with diabetes (type 1 or type 2)? - select all that applies	No
			Yes: grandparent, aunt, uncle or first cousin (but not own parent, brother, sister or own child
			Yes: parent, brother, sister or own child
Prediabetes knowledge	K1	Prediabetes condition can lead to	Type 2 diabetes mellitus*
			Type 1 diabetes mellitus
			Both
			None
			I don’t know
	K2	What is the probability of one developing prediabetes if both their parents have type 2 Diabetes?	0%
			10-15%
			25-40%
			More than 50%*
			I don’t know
	K3	What is the most effective way of identifying prediabetes conditions?	Blood testing*
			Urine testing
			Both
			None of the above
			I don’t know
	K4	What is the fasting blood glucose level (after an overnight fast of 10 hours) in prediabetes?	Less than 100 mg/dL
			100-125 mg/dL*
			140-199 mg/dL
			More than 200 mg/dL
			I don’t know
	K5	Average blood glucose for the past three months is given by the blood test	HbA1c test*
			Fructosamine test
			Fasting blood glucose test
			Oral glucose tolerance test
			I don’t know
	K6	What is the importance of testing insulin levels along with glucose levels in prediabetes?	To identify insulin tolerance
			To identify insulin overdose
			To identify insulin resistance*
			None of the above
			I don’t know
	K7	What is the best advice for those with prediabetes?	Diet control and exercise*
			Insulin injections
			Dental check-up
			Taking medications
			None of the above
			I don’t know
	K8	A person who is prediabetic should regularly consume	Foods that are high in fat
			Soft drinks and energy drinks
			High-fiber foods*
			Foods rich in carbohydrates
			I don’t know
	K9	How often should people with prediabetes exercise?	Most days of the week for at least 30 minutes*
			Once a week for at least 30 minutes
			Once a month for at least one hour
			None of the above
			I don’t know
	K10	How much does weight loss benefit obese patients with prediabetes?	Greatly help*
			Slightly help
			Will not help
			Unsure
Prediabetes attitudes		Please select the degree to which you agree with the following statements:	Likert scale (strongly agree, agree, neither agree nor disagree, disagree, strongly disagree)
	A1	I can do a lot for my prediabetes (to manage it)^#^.	
	A2	Prediabetics should keep their blood sugar close to normal^#^.	
	A3	Control of blood sugar is difficult in prediabetes^$^.	
	A4	There is not much use in blood sugar control in prediabetes because type 2 diabetes mellitus will happen anyway^$^.	
	A5	Prediabetes happens only to an unlucky person^$^.	
	A6	People with prediabetes should be taught about diabetes mellitus^#^.	
	A7	Prediabetes condition is ignored by society^#^.	
	A8	Support from family is important in dealing with prediabetes^#^.	
	A9	Prediabetics should be taught about lifestyle modifications^#^.	
	A10	I can lead a normal life in spite of prediabetes^#^.	
Prediabetes practices	P1	How many hours per week do you perform exercises like cycling, walking, yoga, etc.?	3 to 6 hours a week^†^
			1 to 2 hours a week^§^
			Less than 1 hour a week
			None
	P2	How often do you consume sugar-sweetened beverages (soda, carbonated beverages, and noncarbonated fruit drinks)?	5 or more times a week
			3 or 4 times a week
			1-2 times a week^§^
			Almost never^†^
	P3	How frequently do you substitute fiber-rich foods like oats, whole grains, fruits, or vegetable salads over normal meals?	5 or more times a week^†^
			3 or 4 times a week^§^
			1-2 times a week
			Almost never
	P4	How often do you sleep less than six hours/night?	5 or more times a week
			3 or 4 times a week
			1-2 times a week^§^
			Almost never^†^
	P5	How often do you skip meals?	5 or more times a week
			3 or 4 times a week
			1-2 times a week^§^
			Almost never^†^
	P6	How often do you consume high-fat foods (like fried snacks and meat, fast food, and chocolates)?	5 or more times a week
			3 or 4 times a week
			1-2 times a week^§^
			Almost never^†^
	P7	How often do you eat food while watching TV/using your mobile phone/reading books (distracted eating)?	Every time
			Twice a day
			Once a day^§^
			Almost Never^†^
	P8	How long do you spend in front of a computer/TV in a day?	More than 6 hours a day
			4-6 hours a day
			1-3 hours a day^§^
			Almost Never^†^
	P9	How often do you check your blood sugar at home/lab?	Once in 6 months or yearly
			Once in 2 or 3 months^§^
			Weekly or monthly once^†^
			Never
	P10	How often do you check cholesterol profiles at the lab?	Once in 10 years
			Once in 5 years^§^
			One or more times in 2 years^†^
			Never
Prediabetes video	V1	After submitting your response, you will find a link for a short (two-minute) educational video about prediabetes. Watching the video is optional	I will watch it.
			I will not watch it.
